# Psychological Distress and Concerns of In-Home Older People Living with Cancer and Their Impact on Supportive Care Needs: An Observational Survey

**DOI:** 10.3390/curroncol30110692

**Published:** 2023-10-31

**Authors:** Christos Kleisiaris, Maria Maniou, Savvato Karavasileiadou, Constantinos Togas, Theocharis Konstantinidis, Ioanna V. Papathanasiou, Konstantinos Tsaras, Wafa Hamad Almegewly, Emmanouil Androulakis, Hanan Hamdan Alshehri

**Affiliations:** 1 Department of Nursing, Faculty of Health Sciences, Hellenic Mediterranean University, 71410 Heraklion, Greece; kleisiaris@hmu.gr (C.K.); mariamaniou@hmu.gr (M.M.); harriskon@hmu.gr (T.K.); 2 Department of Community Health Nursing, College of Nursing, Princess Nourah bint Abdulrahman University, Riyadh 11671, Saudi Arabia; skaravasileiadou@pnu.edu.sa; 3 Department of Social Service, Ministry of Public Order, 22100 Tripolis, Greece; o2zcop@otenet.gr; 4 Department of Nursing, University of Thessaly, 41500 Larissa, Greece; iopapathanasiou@uth.gr (I.V.P.); ktsa@uth.gr (K.T.); 5 Department of Statistics and Insurance Science, University of Piraeus, 18534 Piraeus, Greece; mandroulakis@unipi.gr; 6 Department of Medical-Surgical Nursing, College of Nursing, Princess Nourah bint Abdulrahman University, Riyadh 116711, Saudi Arabia; hhalshehri@pnu.edu.sa

**Keywords:** psychological distress, concerns, supportive care needs, cancer survivors, home-based healthcare

## Abstract

(1) Background: Cancer patients are experiencing psychological problems after diagnosis, such as emotional distress and social anxiety, which may increase their demands for emotional and supportive care. This study aimed to assess the influence of both emotional distress and concerns on the supportive care needs of cancer patients receiving home-based healthcare. (2) Methods: In this door-to-door screening program, 97 cancer patients were approached, with a mean age of 73 years old (mean = 73.43; SD = 6.60). (3) Results: As expected, 42.3% of patients highlighted their treatment as their main psychological priority, with 20.6% identifying concerns about the future of their family in this regard. No significant associations with respect to sex were identified in terms of focus, though females reported the need for more frequent psychological support (58.7% vs. 37.3%, respectively, *p* = 0.035) compared to males. Patients who had experienced an increased number of concerns during the last weeks (IRR = 1.02; 95% CI: 1.00–1.03, *p* = 0.007) had a significantly greater risk of presenting an increased rate of supportive care needs. Notably, male patients with bone cancer presented a significantly greater number of supportive care needs (mean rank 45.5 vs. 9.0, *p* = 0.031) respectively, in comparison to those with other types of cancer. (4) Conclusions: Supportive care needs arise from a greater concern and specific type of cancer, highlighting the need for supportive care, such as psychosocial and psychological support. This may have significant implications for treatment and patient outcomes in home care settings.

## 1. Introduction

Cancer is a leading cause of death worldwide, accounting for 10 million deaths in 2020, and nearly one in six of all fatalities. European cases represent 9.7% of all cancers among the global population; yet, this area accounts for 22.8% of all cancer diagnoses and 19.6% of all cancer deaths. Individuals over 65 years old account for 60% of all newly diagnosed malignancies and 70% of all cancer deaths [1]. Among European countries, Greece has attracted attention, as the country ranked the first place of new cancer cases in 2020, in both sexes and all ages, according to the Global Cancer Observatory [2], given that the population over 65 years in Greece is projected to increase from 20% in 2010 to 36% in 2050 [3].

It has been demonstrated that the highest unmet supportive care needs experienced by people with advanced cancer are related to psychological, information, interpersonal, social, physical, family, practical, daily living, spiritual needs, and cognitive needs, irrespective of the type of cancer [4]. In addition, factors associated with higher unmet needs across all domains (information, psychological, physical, etc.) were distress, depression, and anxiety [5] Particularly, both physical and psychological symptoms are common to cancer patients, and 98.7% of cases reported that they felt distressed. Psychosocial concerns are also prevalent in cancer patients and are related to unmet supportive care needs not only during diagnosis and treatment but also across the course of the cancer’s trajectory.

Regarding post-hospital cancer care (home-based care) in Greece, it is underdeveloped and fragmented, with few healthcare services available due to a lack of required planning and coordination. Also, there are no national standards for the operation of palliative care services and clinical guidelines for the provision of palliative care. For example, palliative care services are often provided via non-governmental organizations (NGOs); however, NGOs do not have the authorization to prescribe home care services to patients [6]. Regarding community settings, although psychologists, family physicians, nurses, and social workers (palliative care teams) are able to identify patient and family populations with palliative care unmet needs early at diagnosis, palliative care services are oriented to daily consultations and psychological support due to barriers to delivering the best possible early palliative care approach, such as a lack of education of health professionals on severe pain, grief management, lack of collaboration with experts, and instability of governmental decisions [7]. This highlights the importance of creating new concepts and structures in order to address the psychosocial needs of cancer patients with palliative needs in home care settings [8]. Thus, routine screening for a systematic assessment of patients’ distress and care needs is important for symptom management, continuing counselling, and preparation for the transition from active treatment to the follow-up stages [9].

In Greece, the negative impact of cancer-related anxiety and depression on the quality of life of both patients and caregivers has been well documented in several studies, highlighting the need for appropriate interventions to address psychological distress and manage mental health issues [10,11]. However, to our best knowledge, there are no studies available on cancer-related psychological aspects in terms of the supportive care needs of older patients receiving home-based healthcare services.

Within a framework, health professionals caring for older cancer survivors would benefit from information regarding emotional distress issues and trajectories in supportive care needs and to further comprehend whether these patients might benefit from professional support.

This study was aimed, therefore, to assess the impact of psychological distress and concerns on supportive care needs, taking into consideration patients’ characteristics, existing comorbidities, and the type of cancer of older patients receiving home-based healthcare services.

## 2. Materials and Methods

### 2.1. Study Design

Cancer patients (of any type) who received in-home health services from three “Help at Home” programs in Heraklion City (Crete Island, Greece) were consecutively recruited for a cross-sectional survey for 4 months (between April and July 2016). The ‘Help at Home’ program is supported by the Greek Ministry of Health and operated under the local municipality authorities, providing social care, person-centered nursing, and medical care to their registered members (beneficiaries), mainly to those aged 65 years old and over with chronic disease and disabilities [12].

### 2.2. Sampling and Research Procedures

Since cancer treatment, such as chemotherapy and/or radiotherapy, was not administrated in a home environment, particularly in the “Help at Home” program, but in tertiary hospitals, this constrained the amount of data that could have been collected, such as patients’ medical records and the relationship between the patient and the therapist/oncologist. For this reason, a convenience-sampling method was used, only approaching those with a cancer diagnosis (at least one year) and were registered/beneficiaries of these three “Help at Home” programs. Participants were eligible for participation if they orally consented after being fully informed, were registered members of the “Help at Home” program in Heraklion, were 65 years old or older, had been diagnosed at least one year with any type of cancer (by a public hospital), and were able to understand the questions. Patients with severe psychiatric symptoms, impaired health status, and those who refused to participate without giving any reasons were thus excluded. The required licenses by the respective services (the Municipality of Heraklion) were obtained prior to conducting this survey. Nurses working at these Help at Home programs contacted the patients, explained the study procedures, and prompted the patients to fill out the screening tools.

Of the total of 457 registered members in these (3) homecare programs, a total of 97 out of 109 had a diagnosis of any type of cancer, met the eligibility criteria, and were willing to participate in this door-to-door screening for supportive care needs assessment (response rate: 89%). Accordingly, data of only cancer patients were included in the statistical analysis.

### 2.3. Measures and Data Collection

#### Participant’s Characteristics

In this door-to-door screening program, patients were asked to provide demographic (e.g., sex and age) data and their medical history (e.g., type of cancer, time of diagnosis, and history of chronic diseases), as well as other relevant psychosocial characteristics, such as asking for psychological support. For this data collection procedure, validated screening instruments were applied.

### 2.4. Research Instruments

As far as the assessment of significant emotional distress concerns, the self-assessment questionnaire for patients (SAQ-P) was used. This questionnaire consisted of 14 items rated on a 5-point Likert scale (1 = not at all; 5 = very much). These were used to determine whether and to what extent a patient is in significant distress during the past two weeks that may have required the help of a psychosocial oncology counsellor. A large percentage of answers at ranks 4 or 5 was indicative of a high degree of significant distress. In the present study, only the percentages of answers in the 1-3 range and the 4 or 5 range were thus extracted for descriptive purposes [13]. The mean values, median, and IQR of the SAQ-P FC were calculated and transformed on a scale ranging from 0 to 100).

The self-assessment questionnaire for family and caregivers (SAQ-FC) was also used, which corresponds to the first questionnaire (SAQ-P) with the difference that it refers to the worries and/or concerns of the patient about their support network, such as family members and caregivers. Similarly, it consists of 12 items to varying degrees (not at all to very much), assessing patients’ difficulty dealing with their diagnosis and whether significant distress may be experienced [13]. The mean values, median, and IQR of the SAQ-FC were calculated and transformed on a scale ranging from 0 to 100.

The concern checklist (CCL) was used to assess the different concerns (physical, emotional, etc.) that patients may have had both during the last few weeks (rated on a 5-point Likert scale) and during the last seven days (based on yes/no responses). In this study, answers with ratings of 4 or 5 on the concerns checklist were also considered to indicate a high degree of concern, and these were thus used to present the corresponding descriptive features. In the second part of this questionnaire, referring to patients’ concerns in the last week (LW), the percentage of people who answered “yes” was calculated for each concern. The overall score for the concerns experienced in the last few weeks was then calculated, and the final score was converted to a range from 0 to 100. High mean scores indicated high levels of anxiety. All “yes” answers were summed for each patient to assess the final number of concerns [14].

The original version of the needs evaluation questionnaire (NEQ) was used to identify the supportive care needs of the participants [15]. The NEQ is a self-administered instrument with 23 dichotomous items (Yes/No). It assesses the supportive care needs (needs for support, financial, relational, treatment, communicative needs, etc.) of patients (during the last weeks). The percentages of “yes” answers to each question were thus initially assessed, with further analysis conducted by summing the “yes” answers for each patient and accounted as an actual number. It is worth mentioning that the NEQ was further developed in 2016 and retained the distribution of the items in eight main areas [16]. Then, the Greek version of the NEQ was translated and validated in Greek cancer patients, showing adequate reliability (r = 0.922) by applying the Kuder–Richardson control [17].

### 2.5. Instruments’ Reliability

The instruments were translated into Greek by the main researcher and checked for their reliability. Specifically, to determine whether a collection of items consistently measures the same characteristic, Cronbach’s alpha coefficient was used for the dichotomous items (yes/no), while Kuder–Richardson was applied for the Likert (5-point) items and controlled via the Pearson r and Spearman rs values (*p*-values), respectively. All correlations among instruments demonstrated an adequate internal consistency ‘reliability’ (*p* = 0.001), allowing us to further proceed towards statistical analysis (Supplementary Table S1). The descriptive analysis of the study instruments (e.g., transformed scores, interquartile range, mean values, median, and standard deviation) are presented in (Supplementary Table S2), whereas correlations among screening tools with respect to age and year of diagnosis are presented in (Supplementary Table S3).

### 2.6. Ethical Considerations

The Scientific Committee of the Nursing Department of the Hellenic Mediterranean University ethically approved this study. Permission for the study investigation was sought from the Municipal Enterprise of Culture, Tourism, and Development of Heraklion (D.E.P.T.A.I.) of Heraklion, Crete, Greece (where the “Help at Home” program is based). Prior to data collection, patients were informed in detail about the purposes and procedures of this study. Furthermore, they were assured of the confidentiality of their data and reminded about the voluntary nature of participation. The participants were then asked to give their oral (not written) consent and to complete the questionnaires. The procedure was anonymous, in full compliance with the European Union General Data Protection (Regulation 2016/679 on sensitive personal data) [18].

### 2.7. Data Analysis

Quantitative variables were presented in the form of mean values and standard deviation, while qualitative ones were presented using frequencies and percentages. The Shapiro–Wilk and Shapiro–Francia statistical tests were used on a case-by-case basis to assess the normality of quantitative variables across this study. The existence of normality was also confirmed or rejected by means of visual oversight of the corresponding histograms, the normal Q–Q plot diagrams, and the box-plots. The median and interquartile range (intermediate and IQR, respectively) were used as descriptive measures where normality in the data was rejected. Exact tests were applied to identify possible relations among patients’ characteristics and the transformed scores of the tools in cases considered appropriate. Monte Carlo simulations (with 10,000 replications) were also used in cases considered appropriate. The chi-squared (χ^2^) test was used to identify associations between males and females. Statistical methods (including the *t*-test, Mann–Whitney U test, and Kruskal–Wallis test) were used to explore possible significant differences among qualitative variables (e.g., tools’ scores, sex-related differences, and type of cancer based on organ system) in cases where comorbidities were present. Yet, Spearman’s rho correlation coefficient was also used to analyze the quantitative variables (e.g., age and year of diagnosis). To explore our main research question, whether psychological distress and concerns (including confounding variables) exhibit negative or positive effects on supportive care needs, the NEQ was placed as a dependent variable. However, since supportive care needs (NEQ items) were estimated as counts, the Poison regression model was considered more appropriate. However, due to over-dispersion, as confirmed via a likelihood ratio test of alpha = 0: G2 = 191.07 (*p*-value < 0.001), a standard negative binomial regression model was considered as the most appropriate along with adjusted robust standard errors, even after examination for the model of zero-inflated negative binomial regression, exploring the hypothesis of ‘excessive zeros’ using Vuong’s test (*p* > 0.05). Statistical analysis was performed using IBM SPSS 20.0 and STATA MP 13, with a *p*-value set to <0.05.

## 3. Results

### 3.1. Demographical Characteristics

Most respondents (52.6%) were men, and their mean age was 73.4 years (SD = 6.60). The mean time from diagnosis was 3.15 years, and the most common type of cancer using grouping based on anatomical systems was lymphatic cancer (*n* = 25). The next most common cancers were respiratory cancer (*n* = 23) and digestive cancer (*n* = 20). Finally, only a few patients (21%) received psychological support (Table 1).

### 3.2. Distress Level Experienced by the Participants during the Past Two Weeks

Table 2 illustrates the levels of significant distress (4–5) experienced by the participants during the past two weeks. About 53.6% of respondents reported feeling significantly more depressed or discouraged, and 61.9% reported that their diagnosis of cancer has caused financial hardship to their family. However, no significant associations were observed between males and females regarding significant distress (Table 2). However, significant distress differences (SAQ-P) were observed in patients suffering from chronic diseases compared to those free of chronic disease (mean rank: 51.14 vs. 33.83, *p* = 0.036), respectively, Mann–Whitney U = 328.00, *p* = 0.036), suggesting that chronic diseases are associated with higher rates of significant distress (Supplementary Table S4).

Table 3 presents patients’ distress levels experienced concerning family and caregivers during the past two weeks. No significant differences between males and females were observed regarding the presence of significant distress (4–5) (Supplementary Table S5).

### 3.3. Sex Differences with a Strong Feeling of Psychological Concern

In Table 4, sex differences with a strong feeling of psychological concern during the last month (LM) and the last week (LW) were presented. Respondents identified four main concerns that were of greatest concern to them during the LM. About 64.9% of respondents reported that they were very concerned about their treatment, while the same percentage reported being very concerned about how they physically felt. The study results’ further indicated that 61.9% of respondents were very concerned about the illness itself, while 58.8% of respondents were very worried about their finances. Similarly, 58.8% of respondents reported feeling upset or distressed as one of their important concerns. However, no associations were observed between males and females regarding their strong feelings of psychological concern during the last month (LM).

The major concern identified during the last week (LW) was about their treatment (85.6%), while 64.9% of respondents reported concerns about their current illness. In addition, 60.8% of the respondents reported being very concerned about their finances. Around half (53.6%) were concerned about how they felt physically, and 46.46% of respondents reported a strong feeling of worrying about the future as their most important concern. Similarly, no associations were observed between males and females regarding their strong feelings of psychological concern during the last week. However, significant differences in concern during the last month (CC-LM) were observed between patients suffering from comorbidities and those free of comorbidities (mean rank 51.15 vs. 33.79, respectively, Mann–Whitney U = 327.5, *p* = 0.042), suggesting that comorbidities are associated with a higher feeling of concern. In addition, significant differences in concern during the last week (CC-LW) were observed between patients suffering from comorbidities and those free of comorbidities (mean rank 51.22 vs. 33.29, respectively, Mann–Whitney U = 321.5, *p* = 0.037), suggesting that existed comorbidities significantly increased the feeling of concerns in the last week (Supplementary Table S6).

### 3.4. Sex Differences in the Supportive Care Needs

In Table 5, a variety of cancer-related needs, linked to the condition of one’s health, which people have said they have had, is presented. Patients reported higher levels of a need “to be more involved in the therapeutic choices” (67%), and “to have more information about my future condition” (61.9%). They also reported that they needed “more explanations of treatments” (58.8%). In contrast, about 23.7% of patients reported lower levels of needs in terms of better respect for their intimacy, while 25.8% reported a need to feel less abandoned and “to respect for my intimacy” (23.7%). Moreover, 32.0% of respondents reported they needed to receive “less commiseration from other people.” Identifying their foremost need at this moment, 42.3% of patients expressed a foremost need for “treatment progress”, “future condition”, “family” (20.6%), and “financial aspects” (16.5%).

Finally, no significant associations were found between males and females across all items concerning supportive care needs, apart from the need “to speak with a psychologist” (females 58.7 vs. males 47.4, *p* = 0.035). (Supplementary Table S7)

### 3.5. The Effect of Significant Emotional Distress and Concerns on the Supportive Care Needs

Table 6 refers to the influence of significant distress, with concerns including comorbidities, age, sex, and years of diagnosis (independent variables) on supportive care needs of the study respondents during the last weeks, using the NEQ as a dependent variable. It is mentioned that patients’ supportive care needs significantly increased when patients experienced stronger feelings of concern during the last month (CC-LM). Specifically, patients’ concerns that increased by one unit resulted in an increased rate of supportive care needs by a factor of 1.022 (IRR = 1.022; 95% CI: 1.005–1.038; *p* = 0.007) independently of other variables. However, this association was not significant for concerns during the last week (IRR = 1.013; *p* = 0.672).

Moreover, patients who received psychological support were presented with a greater likelihood of reducing their rate of needs by a factor of 0.682 (IRR = 0.682; 95% CI: 0.485–0.958, *p* = 0.027) compared to those who did not receive psychological support, although females significantly reported more frequently “to speak to a psychologist” compared to males (58.7% vs. 37.3%), respectively. No other significant associations were found with respect to an increased rate of supportive care needs (IRR = 0.716; 95% CI: 0.411–1.248; *p* = 0.240) among patients with comorbidities (yes vs. no). Demographic characteristics suggested that comorbidities, age, sex, years of diagnosis, and significant distress are not risk factors for an increased number of supportive care needs in our sample. In addition, significant differences in supportive care needs (NEQ) among males were observed with respect to the organ system, suggesting that male patients suffering from bone cancer (impacting the skeletal system) presented with higher supportive care needs compared to patients suffering from other types of cancer (Supplementary Table S8).

## 4. Discussion

This study aimed to investigate whether significant distress and concerns may affect patients’ supportive care needs. Our data analysis showed that patients’ supportive care needs significantly increased when patients experienced stronger feelings of concern during the last month, whereas this association was not significant for feelings of concern experienced in the past week and when patients experienced significant distress. In addition, patients suffering from bone cancer were presented with higher supportive care needs compared to those suffering from other types of cancer. Finally, patients who had received psychological support were presented with a greater likelihood of reducing the number of needs by 0.682 times compared to those who did not receive psychological support. No other significant effects on supportive care needs were observed.

The main finding of the present study was that the supportive care needs of cancer survivors are significantly affected by long-term concerns and/or worries (but not significant distress), especially about their treatment progress. In comparison to our findings, the results of the current study conducted in a Greek sample of 86 patients newly diagnosed exploring the unmet care needs, social support, and distress from the initial diagnosis to post-surgery in gynecological cancer showed that changes in anxiety levels were associated with changes in needs related to family, need for assistance/care and support, and total needs. However, in agreement with our findings, the overall level of distress did not significantly change from the first post-operative follow-up visit, and 4 months after surgery [19]. Yet, our study sample consisted of both sexes and various types of cancer, and, therefore, any comparison ought to consider several other factors, such as the age, the stage, and the type of cancer. On the contrary, a similar study conducted on Greek patients with gynecological cancer reported that increased care needs pre-operatively are associated with their psychological distress, despite the high levels of social support that they have received [20]. Possible explanations for these differences could be that the distress level was assessed with the use of the DASS-21 scale, and therefore the prevalence of stress or the statistical methods used may significantly differ in comparison to SAQ-P scoring and our analyses in general. Along the same lines, higher levels of supportive care needs were associated with higher levels of intrusive thoughts about cancer among lung cancer survivors, especially those who reported higher physical distress [21]. The effect of symptoms and psychological distress on supportive care needs is extensive in lung cancer patients [22]. In our study, we found no significant differences in distress and strong feelings of concern between males and females with respect to the type of cancer or organ system apart from bone cancer. Across other types of cancer, findings of a cross-sectional study among 450 breast cancer survivors showed that one in four women presented cognitive–emotional distress related to unmet psychosocial needs; however, there was a considerable level of need, even among long-term survivors [23]. A recent prospective cohort study [24], aimed to describe patients’ self-reported distress trajectory over time and how this was associated with their wellbeing, and supportive care needs among brain cancer patients, demonstrated that older participants consistently reported higher distress levels in comparison to younger participants, whereas high distress trajectory participants had less education, a lower physical wellbeing, and more unmet needs. Most likely, older patients experience a major change in their relationship with the doctors once the intensive treatment period is over. For instance, frequently postponed follow-up sessions because they were no longer at the top of the doctor’s priorities and/or contact with many doctors for various treatments at various hospitals, and thus higher levels of emotional distress or concerns may be experienced by older cancer survivors, highlighting the unique role of nursing in improving the quality of care for cancer survivors [25]. Remarkably, in our study, older patients suffering from comorbidities experienced higher levels of significant distress compared to those free of comorbidities, although comorbidities were not associated with the increased number of supportive care needs, suggesting that chronic diseases are associated with higher rates of significant distress. However, our study screened various types of cancer in home care; thus, this should be considered when interpreting and comparing these results to other studies. For example, regardless of cancer disease, the elderly population experienced higher levels of cognitive and physical declines (depression, anxiety, and physical limitations disabilities in activities of daily living), and therefore higher levels of psychological distress and concern may coexist (overlap) with cancer-related psychological issues in these patients [26]. It is worth mentioning that cancer care has been profoundly impacted by the global pandemic of COVID-19, placing numerous challenges [27]. For example, older adults living with dementia in the COVID-19 world have experienced reduced access to support and activities. These changes have caused distress and exacerbated behavioral and psychological symptoms of dementia [28]. However, our data was collected in 2016, suggesting that this should be considered when interpreting and comparing the results to post-COVID-19 studies.

Furthermore, it has been found that patients suffering from bone cancer are presented with higher supportive care needs compared to those suffering from other types of cancer. Possible explanations for these associations could be attributed to recovering from bone cancer and adjusting to life after treatment is different for each person, depending on long-term side effects and concerns, such as rehabilitation after bone cancer surgery, self-esteem body image, and sexuality [29].

Another important finding was that accepting psychological support reduces the perceived number of supportive needs, although only 21.6% had received psychological support, and less than half of the patients were willing to accept help for psychological or emotional difficulties. Konstantinidis and his colleagues [17], demonstrated similar findings in a recent Greek study, having recorded the needs of patients and their levels of desire for psychological help, as well as having assessed the psychosocial interventions that have already been implemented, reported that there is no high need for discussions with a spiritual advisor. The results showed that where patients received psychological support, the perceived number of needs decreased [30]. Overall, women expressed a greater desire to receive psychological support than men, while most oncology patients did not receive any psychological support [31]. In the current study, females demonstrated a greater desire for psychological support than men. Weis et al. (2018), who justified that men tend to be more socially restrained and less willing to discuss emotional and psychological issues, reported similar results [32]. This tendency has been attributed to the traditional position of men in many societies, where in admitting to weakness and acknowledging the need for help are not believed to correspond to the characteristics of the gender [33]. Finally, most patients did not receive sufficient psychological support, even though many of them would have liked to receive some kind of help in this manner. This finding reflects the studies reporting high rates of willingness to accept psychological support especially during the quarantine (COVID-19) period [27]. Notably, under Greek community healthcare settings, the provision of psychological support is limited due to a lack of education and communication with experts and families [7].

The primary need identified by oncology patients was treatment, followed by reassurance about the future and their families, and then finances. The results of other studies have not identified patients having similar needs, and, in contrast to the current results, previous research has shown that the greatest needs among such patients include managing emotions, accepting death based on religious beliefs, and then managing finances [34]. Recovery of functionality was also identified as the most important need in another study, followed by reassurance about the future, and then treatment [35].

### 4.1. Future Implications

The findings of this study demonstrate the need for home care agencies and healthcare organizations to collaborate to improve care by implementing shared evidence-based practice recommendations that guarantee person-centered care for elderly cancer patients in home care settings. Patient-centered home care is the key model of care in terms of improving the quality of life for elderly cancer patients, and this must be delivered by experienced multidisciplinary teams. Practically, psychosocial interventions are effective in reducing distress in cancer patients [36,37], and even a brief intervention is likely to be superior to conventional care in terms of reducing the perceived number of unmet needs, as well as their urgency. Support interventions should usefully integrate lifestyle issues, neuropsychological rehabilitation, and coping support. In agreement with other studies for nursing practice, it is recommended to expand the role of oncology nurses, providing them with the training and competencies needed to formally declare them as care managers throughout the continuum of cancer care [25].

### 4.2. Limitations

The most important limitation of the present research is the representativeness of the empowered sample used for this study. In addition, validity and psychometric tests were not performed to confirm the theoretical factor structure of the measurements for the Greek population, although all instruments used demonstrated adequate reliability for our sample. Another important limitation is that the data were drawn from a small sample size and regional focus; thus, the generalization of our findings compared to other national-based population cohort surveys should be made with caution. For future research, it is recommended to recruit a representative considerable number of participants across different Greek regions. In addition, the methodological approach used may not have been the most appropriate in this case due to the requirements of qualitative research. Also, homecare settings have no access to the medical records of each patient, as the treatment is only administrated in hospitals. Hence, we were unable to provide information regarding the stage of each type of cancer due to resource constraints [38]. As the disease was not staged in this case, the results may vary were a different sample to be used, as the relevant needs may increase depending on the stage. Moreover, the patient’s annual income was not recorded to account for financial needs, as this was not seen as being allied with the purpose of the research. Several psychological variables, such as anxiety, depression, and financial aspects, were not considered in the current study that may be connected to distress levels and thus increase the need for psychological support. Further studies should seek to incorporate an evaluation of the financial expenses of diagnosis and treatment, as financial struggles caused by such issues have been linked to lower levels of mental health in cancer survivors and could enhance the stressful nature of living with such illnesses. Finally, although the data was collected in 2016, it is important to further investigate the role of COVID-19 and the needs of cancer patients, especially in the emotional and psychological aspects due to existential anxieties.

## 5. Conclusions

The data suggest that cancer patients who experienced higher levels of long-term psychosocial concerns are associated with an increased number of supportive care needs, highlighting the need for professional support to decrease. Therefore, it is crucial to enhance the training and capacities of health professionals, including oncology nurses working in home-based healthcare, by incorporating specific skills and evidence-based practice recommendations related to palliative care into their daily practice.

## Figures and Tables

**Table 1:** Participants’ characteristics (*n* = 97).

Variable		Mean (SD)	Median (IQR)
Age (years) *		73.43 (6.60)	72.00 (9.50)
Diagnosis (years) *		3.15 (3.47)	2.00 (3.50)
		*n* = 97	%
Sex	Male	51	52.6
	Female	46	47.4
Type of cancer	Breast	15	15.5
	Lung	13	13.4
	Prostate	12	12.4
	Larynx	10	10.3
	Colon	9	9.3
	Adenoid (lymph nodes)	7	7.2
	Bladder	7	7.2
	Bone	5	5.2
	Ovary	5	5.2
	Pancreas	4	4.1
	Thyroid	3	3.1
	Stomach	3	3.1
	Esophagus	3	3.1
	Anus	1	1.0
Type of cancer (grouped into organ system)	Reproductive	5	5.2
	Urinary	19	19.6
	Digestive	20	20.5
	Respiratory	23	23.7
	Lymphatic	25	25.8
	Skeletal	5	5.2
Most prevalent comorbidities	Hypertension	48	56.5
	Diabetes mellitus	31	36.5
	Osteoporosis	22	25.9
	COPD	17	20.0
	Arthritis	15	17.6
Living with the family	Yes	91	78.4
	No	6	5.2
Received psychological support	Yes	21	21.6
	No	76	78.4

Abbreviation: COPD—chronic obstructive pulmonary disease. * Note: Years of Diagnosis: the time of period after the diagnosis of cancer that the data collected.

**Table 2:** Patients’ significant distress differences according to sex.

	Item	1–3	4–5	4–5 (%)		*p*-Value
		%	%	Female	Male	
	During the last 2 weeks:					
1	I have felt anxious or worried about cancer and the treatment I am receiving.	48.5	51.5	45.7	56.9	0.270
2	I have felt depressed or discouraged.	46.4	53.6	52.2	54.9	0.788
3	I have been irritable or unusually angry and I have not controlled it well.	69.1	30.9	32.6	29.4	0.734
4	My sleeping habits have changed.	61.9	38.1	37.0	39.2	0.819
5	I have experienced a change in my appetite.	58.8	41.2	43.5	39.2	0.670
6	I have had difficulty concentrating at work, home, or on routine things, such as reading the newspaper or watching TV.	70.1	29.9	34.8	25.5	0.318
7	Cancer and its treatment have interfered with my daily activities.	49.5	50.5	45.7	54.9	0.363
8	Cancer and its treatment have interfered with my family and social life.	66.0	34.0	32.6	35.3	0.780
9	Cancer and its treatment have interfered with my sexual life.	94.8	5.2	0.0	9.8	0.058
10	Pain and discomfort have caused me to limit my activities.	47.4	52.6	50.0	54.9	0.629
11	Cancer has caused physical, emotional, or financial hardships for me.	38.1	61.9	56.5	66.7	0.304
12	Cancer and its treatment have caused changes in my physical appearance, and this concerns me.	56.7	43.3	45.7	41.2	0.657
13	I have had difficulty coping with the stress I have experienced.	62.9	37.1	37.0	37.3	0.976
14	My quality of life during the past two weeks has been: rate: from excellent (1–3) to very poor (4–5).	52.6	47.4	42.2	52.0	0.341

Note: patients’ responses are based on the self-assessment questionnaire for patients (SAQ-P), which is a 5—point screening tool (ranging from 1 to 5); patients’ responses (1–3) indicate decreased feelings of distress and (4–5) a significant feeling of distress. Statistical method: the chi-squared test (χ^2^).

**Table 3:** Sex differences in significant distress with respect to family and caregivers.

	Item	1 or 3	4 or 5	4–5 (%)		*p*-Value
		%	%	Female	Male	
1	I feel anxious or worried about my loved one’s cancer diagnosis/treatment.	38.0	62.0	65.1	59.2	0.559
2	I feel depressed or discouraged.	48.9	51.1	44.2	57.1	0.215
3	I have been irritable or unusually angry and have not controlled it well.	68.5	3.5	34.9	28.6	0.516
4	My sleep habits have changed.	58.7	41.3	39.5	42.9	0.747
5	I have experienced a change in my appetite.	66.3	33.7	30.2	36.7	0.510
6	I have difficulty concentrating at work, home, or school, or on routine things, such as reading the newspaper or watching TV.	76.1	23.9	23.3	24.5	0.890
7	My loved one’s diagnosis/treatment interferes with my daily activities.	47.8	52.2	44.2	59.2	0.151
8	My loved one’s diagnosis/treatment interferes with my family or social life.	55.4	44.6	41.9	46.9	0.625
9	My loved one’s diagnosis/treatment interferes with my sexual life.	89.1	10.9	14.0	8.2	0.506
10	My loved one’s diagnosis has caused financial hardship to our family.	35.9	64.1	58.1	69.4	0.262
11	I have difficulty keeping up with my caregiving activities.	51.1	48.9	48.8	49.0	0.989
12	I have difficulty coping with the stress the entire family is experiencing.	57.6	42.4	41.9	42.9	0.923

Note: patients’ responses as regards distress about family and caregivers based on the self-assessment questionnaire for family and caregivers (SAQ-FC), which is a 5—point screening tool (ranging from 1 to 5); patients’ responses (1–3) indicate a decreased feeling of distress and (4–5) a significant feeling of distress. Statistical method: the chi-squared test (χ^2^).

**Table 4:** Sex differences with a strong feeling of psychological concern during the last month (LM) and the last week (LW).

	Item	4 or 5 (%)	4 or 5 (%)		*p*-Value
			Female	Male	
	During the last month (LM)				
1	The illness itself (what is it, is it better, etc.).	61.9	63.0	60.8	0.819
2	How I have been feeling physically.	64.9	60.9	68.6	0.424
3	Treatment for the illness.	64.9	63.0	66.7	0.709
4	Feeling different from other people.	25.8	28.3	23.5	0.595
5	Feeling upset or distressed.	58.8	54.3	62.7	0.402
6	Not being able to do the things I used to do.	44.3	47.8	41.2	0.510
7	The future.	49.5	43.5	54.9	0.261
8	My job.	16.5	15.2	17.6	0.747
9	Finances.	59.8	56.5	62.7	0.532
10	My relationship with my partner.	37.1	37.0	37.3	0.976
11	My relationship with others.	27.8	28.3	27.5	0.929
12	How I feel about myself as a man or woman.	10.3	10.9	9.8	0.863
13	Support I have.	39.2	45.7	33.3	0.215
14	Any other concerns? Please describe:	-			-
	**Item**	**Yes (%)**	**Yes (%)**		***p*-Value**
			**Female**	**Male**	
	In the last 7 days (LW) have you been worrying about:				
1	Your current illness?	64.9	63.0	66.7	0.709
2	How you are feeling physically?	53.6	63.0	45.1	0.077
3	Your treatment?	85.6	87.0	84.3	0.712
4	Feeling different from other people?	22.7	23.9	21.6	0.783
5	Feeling upset or distressed?	46.4	41.3	51.0	0.340
6	Not being able to do the things that you used to do?	30.9	28.3	33.3	0.589
7	The future?	46.4	41.3	51.0	0.340
8	Your job?	9.3	10.9	7.8	0.732
9	Your finances?	60.8	67.4	54.9	0.208
10	Your relationship with your partner?	41.2	41.3	41.2	0.990
11	Your relationship with other people?	22.7	26.1	19.6	0.447
12	How you feel about yourself as a man/woman?	8.2	8.7	7.8	0.879
13	The support you are receiving?	19.6	23.9	15.7	0.308
14	Are there any other concerns that have not been mentioned?	0.0	-	-	-

Note: patients’ responses based on the CC-LM and CC-LW. The concern checklist-last month is a 5-point screening tool (ranging from 1 to 5); patients’ responses: from not a worry to extremely worried; (1–3) indicate a decreased feeling of concern, while (4–5) indicate a strong feeling of concern; CC-LM: Patients were asked about different concerns that that they may have been worried about regarding their illness and treatment over the last month; CC-LW: Patients were asked about specific problems that they may have worried about. If the answer was “yes” the specific concern was explored. For each problem, it was established whether it was small, medium, or large; Statistical method: the chi-square test (χ^2^); each ‘Yes’ was accounted as “1 concern” and estimated as an actual number.

**Table 5:** Sex differences in the supportive care needs of the study participants.

	NEQ Item	Yes (%)	Yes (%)		*p*-Value
			Female	Male	
1	I need more information about my diagnosis.	50.5	54.3	47.1	0.473
2	I need more information about my future condition.	61.9	65.2	58.8	0.517
3	I need more information about the exams I am undergoing.	49.5	54.3	45.1	0.363
4	I need more explanations of treatments.	58.8	63.0	54.9	0.416
5	I need to be more involved in the therapeutic choices.	67.0	71.7	62.7	0.347
6	I need clinicians and nurses to give me a more comprehensible Information.	39.2	34.8	43.1	0.414
7	I need clinicians to be more sincere with me.	51.5	47.8	54.9	0.486
8	I need to have a better dialogue with clinicians.	48.5	52.2	45.1	0.486
9	I need my symptoms (pain, etc.) to be better controlled.	56.7	58.7	54.9	0.707
10	I need more help with eating, dressing, and going to the Bathroom.	36.1	39.1	33.3	0.553
11	I need better respect for my intimacy.	23.7	21.7	25.5	0.664
12	I need better attention from nurses.	39.2	34.8	43.1	0.414
13	I need to be more reassured by the clinicians.	52.6	58.7	47.1	0.252
14	I need better services from the hospital (bathrooms, etc.).	39.2	30.4	47.1	0.094
15	I need to have more economic insurance information (tickets, invalidity, etc.) in relation to my illness.	54.6	58.7	51.0	0.446
16	I need economic help.	47.4	50.0	45.1	0.629
17	I need to speak with a psychologist.	47.4	58.7	37.3	0.035
18	I need to speak with a spiritual advisor.	36.1	43.5	29.4	0.150
19	I need to speak with people who have this same experience.	52.6	58.7	47.1	0.252
20	I need to be more reassured by my relatives.	47.4	54.3	41.2	0.195
21	I need to feel more useful within my family.	45.4	52.2	39.2	0.201
22	I need to feel less abandoned.	25.8	21.7	29.4	0.388
23	I need to receive less commiseration from other people.	32.0	30.4	33.3	0.760
24	At this moment, my foremost need is:		%	%	
	Treatment progress;	42.3	45.7	39.2	0.131
	Future condition;	20.6	28.3	13.7	
	Financial aspects;	16.5	10.9	21.6	
	Family.	20.6	15.2	25.5	

Note: A positive answer (yes) to at least one question in each needs category was coded as a positive response to the respective category, accounted as a “1 need” and estimated as an actual number based on the needs evaluation questionnaire (NEQ). Statistical method: the chi-squared test (χ^2^).

**Table 6:** The effect of significant emotional distress and concerns on the supportive care needs of the study participants.

NEQ	IRR	S.E	z-Value	*p*-Value	95% C.I	
SAQ-P	1.001465	0.007016	0.21	0.834	0.9878082	1.015311
CC-LM	1.022063	0.0082974	2.69	0.007	1.005929	1.038455
CC-LW	1.013672	0.0324882	0.42	0.672	0.9519555	1.079391
Sex (male vs. female)	0.9123177	0.1569902	−0.53	0.594	0.6511375	1.278261
Comorbidities (yes vs. no)	0.7165757	0.2031238	−1.18	0.240	0.4111277	1.248957
Psychological Support (yes vs. no)	0.6817649	0.1184455	−2.20	0.027	0.4850119	0.9583341
Age (years)	0.9999161	0.0162301	−0.01	0.996	0.9686063	1.032238
Diagnosis (year)	0.990662	0.0255254	−0.36	0.716	0.9418753	1.041976
lnalpha	−0.4975616	0.2396869			−0.9673392	−0.027784
Alpha	0.6080114	0.1457323			0.380093	0.9725984

Abbreviations: SAQ-P = self-assessment questionnaire for patients; CC-LM: concern checklist- last month; CC-LW: concern checklist-last week; IRR: incidence rate ratio; C.I: confidence interval; and S.E: standard error. Note: Statistical method: Standard negative binomial regression was confirmed as the most appropriate regression model (likelihood ratio test of alpha = 0: G2 = 191.07, *p*-value < 0.001), with the Vuong’s test applied for possible excessive zeros (*p* > 0.05). The NEQ was used as a dependent variable. The scores of the CC-LW and NEQ were referred to as the total number of concerns and needs, ranging 1–13 and 0–23, respectively. The scores of the CC-LM and SAQ-P were presented as transformed scores ranging from 0 to 100.

## Data Availability

The data presented in this study are available on request from the corresponding author. The data are not publicly available due to privacy restrictions.

## Supplementary Materials

The following supporting information can be downloaded at: www.mdpi.com/xxx/s1,Table S1: Internal consistency and correlations among instruments; Table S2: Descriptive analysis of the study instruments; Table S3: Correlations among screening tools with respect to age and year of diagnosis; Table S4: Sex differences in significant distress with respect to the type of cancer; Table S5: Sex differences in significant distress about family and caregivers with respect to the type of cancer; Table S6: Sex differences in concerns during the last month with respect to the type of cancer; Table S7: Sex differences in concerns during the last week with respect to the type of cancer; Table S8: Sex differences in supportive care needs with respect to the type of cancer.
