# Supplementary material for: Psychological Distress and Concerns of In-Home Older People Living with Cancer and Their Impact on Supportive Care Needs: An Observational Survey
Source: Curr Oncol. 2023 Oct 31;30(11):9569–83. doi: 10.3390/curroncol30110692 (PMC10670276; doi:10.3390/curroncol30110692)
Supplement: Supplementary file 1 [file curroncol-30-00692-s001.zip › curroncol-2636254-supplementary.pdf]

Table S1. Internal consistency and correlations among instruments (Cronbach's alpha)

	Self–Assessment Questionnaire for Patients (SAQ-P)	*Self–Assessment Questionnaire for family and caregivers (SAQ-FC)*	*Concerns Checklist - last month (CC-LM)*	*Concerns Checklist - last week (CC-LW)*	*Needs Evaluations Questionnaire (NEQ)*
Self–Assessment Questionnaire for Patients (SAQ-P)	(0.89)				
Self–Assessment Questionnaire for family and caregivers (SAQ-FC)	r=0.82 (<0.001)	(0.93)			
Concerns Checklist - last month (CC-LM)	r=0.74 (<0.001)	r=0.68 (<0.001)	(0.90)		
Concerns Checklist - last week (CC-LW)	r_s=0.53 (<0.001)	r_s=0.48 (<0.001)	r_s=0.76 (<0.001)	(0.77)	
Needs Evaluations Questionnaire (NEQ)	r_s=0.47 (<0.001)	r_s=0.47 (<0.001)	r_s=0.59 (<0.001)	r_s=0.49 (<0.001)	(0.95)

Table S1 Methodological clarifications:

For the reliability of the instruments, Cronbach's alpha coefficient was used for the dichotomous items (scales), while Kuder-Richardson (in parenthesis) for the Likert (5-point) items as shown in a diagonal line and is explored by the Pearson-r and Spearnman-rs (p-values), respectively.

All correlations among instruments showed adequate reliability (p=0.001) allowing us to further proceed to statistical analysis.

Table S2. Descriptive analysis of the study instruments

Instruments	Mean	S.D	Median	IQR	Range
Self–Assessment Questionnaire for Patients (SAQ-P)	51.09	22.03	51.11	36.67	0-100
Self–Assessment Questionnaire for family and caregivers (SAQ-FC)	52.46	24.63	52.63	3.47	0-100
Concerns Checklist - last month (CC-LM)	58.85	23.07	58.00	39.00	0-100
*Concerns Checklist last week (CC-LW) **	5.12	2.93	5.00	4.00	1-13
*Needs Evaluation Questionnaire (NEQ)**	10.63	7.89	11.00	16.00	0-23

*Transformed scores (0-100); IQR=interquartile range

*Note that CC-LW (13 items) and NEQ (23 items) scores are accounted as actual numbers – summing of the dichotomous items "Yes" (positive responses).

Table S3. Correlations among screening tools with respect to age and year of diagnosis.

Tools	Transformed scores (0-100)	
	**Age**	**Year of diagnosis**
SAQ-P	r_s=0.051, (0.623)	r_s=0.031, (0.761)
SAQ-FC	r_s= 0.076, (0.469)	r_s=0.136, (0.195)
CC-LM	r_s= 0.051, (0.620)	r_s=0.050, (0.629)
CC-LW	r_s=0.055, (0.593)	r_s= -0.076, (0.462)
NEQ	r_s=0.008, (0.940)	r_s= -0.177, (0.083)

*Transformed scores (0-100); Spearnman rs confidence; (p-values is presented in parentheses)

Table S4. Sex differences in significant distress with respect to the type of cancer (Kruskal-Wallis test)

Sex		Organ system	Mean Rank	Test statistics
Female	SAQ-P	Reproductive	19.70	$\chi^2(5)=3.052$, p-value=0.724
		Urinary	33.83	
		Digestive	27.00	
		Respiratory	23.38	
		Lymphatic	21.71	
		Skeletal	24.50	
Male	SAQ-P	Urinary	26.78	$\chi^2(4)=7.580$, p-value=0.102
		Digestive	23.23	
		Respiratory	26.67	
		Lymphatic	15.13	
		Skeletal	45.00	

Table S5. Sex differences in significant distress about family and caregivers with respect to the type of cancer (Kruskal-Wallis test)

Sex		Organ Systems	Mean Rank	Test statistics
Female	SAQ-FC	Reproductive	17.00	$\chi^2(5)=4.832$, p-value=0.462
		Urinary	29.17	
		Digestive	19.29	
		Respiratory	25.00	
		Lymphatic	20.90	
		Skeletal	35.25	
Male	SAQ-FC	Urinary	26.57	$\chi^2(4)=5.277$, p-value=0.260
		Digestive	25.92	
		Respiratory	22.00	
		Lymphatic	14.50	
		Skeletal	38.67	

Table S6. Sex differences in concerns during the last month with respect to the type of cancer (Kruskal-Wallis test)

Sex		Organ Systems	Mean Rank	Test statistics
Female	CC-LM	Reproductive	18.20	$\chi^2(5)$=1.928, p-value=0.879
		Urinary	29.17	
		Digestive	27.07	
		Respiratory	23.81	
		Lymphatic	22.57	
		Skeletal	24.25	
Male	CC-LM	Urinary	26.28	$\chi^2(4)$=8.586, p-value=0.059
		Digestive	25.12	
		Respiratory	26.03	
		Lymphatic	12.75	
		Skeletal	45.83	

Table S7. Sex differences in concerns during the last week with respect to the type of cancer (Kruskal-Wallis test)

Sex		Organ System	Mean Rank	Test statistics
Female	CC-LW	Reproductive	18.10	$\chi^2(5)$=3.363, p-value=0.680
		Urinary	19.17	
		Digestive	18.43	
		Respiratory	26.56	
		Lymphatic	26.02	
		Skeletal	22.50	
Male	CC-LW	Urinary	28.56	$\chi^2(4)$=8.844, p-value=0.056
		Digestive	21.92	
		Respiratory	28.00	
		Lymphatic	10.75	
		Skeletal	40.33	

Table S8. Sex differences in supportive care needs with respect to the type of cancer (Kruskal-Wallis test)

Sex		Organ System	Mean Rank	Test statistics
Female	NEQ	Reproductive	15.20	$\chi^2(5)$=3.478, p-value=0.653
		Urinary	27.83	
		Digestive	25.14	
		Respiratory	28.25	
		Lymphatic	22.48	
		Skeletal	23.75	
Male	NEQ	Urinary	29.53	$\chi^2(4)$=9.861, p-value=0.031
		Digestive	28.27	
		Respiratory	22.17	
		Lymphatic	9.00	
		Skeletal	39.17	

Tables S4-8. Methodological clarifications and interpretations:

A greater Mean Rank is indicative of a greater instrument's total score of both: dichotomous (Yes/No) and/or transformed scores (0-100).

Types of cancers were grouped according to organ systems to identify possible correlations due to a small sampling size.

Differences in instruments scores with respect to sex (Data not shown in Tables 4-8):

- No significant distress differences (SAQ-P) were observed with respect to sex (t-test, Female: 50.29±23.11 vs Male: 51.81±21.21, t(95)= -0.337, p=0.737). [Table S4]
- No significant distress differences (SAQ-FC) were observed with respect to sex (t-test, Female: 50.18±26.05 vs Male: 54.46±23.39, t (90)= -0.829, p=0.409). [Table S5]
- No significant differences in concerns during the last month (CC-LM) were observed with respect to sex (t-test, Female: 60.00±22.90 vs Male: 57.80±23.39, t (95)= 0.466, p=0.642). [Table S6]
- Also, no significant differences in concerns during the last week (CC-LW) were observed with respect to sex (Mann-Whitney U=1143.00, p=0.827, mean rank for Females=49.65 vs. Males=48.41). [Table S7]
- No significant differences in supportive care needs (NEQ) were observed with respect to sex (Mann-Whitney U=1094.5, p=0.570, mean rank: Females=50.71 vs. Males =47.46).
- Significant differences in supportive care needs (NEQ) among males were observed with respect to organ system (Table S8), suggesting that male patients suffering from bone cancer (Skeletal system) presented with higher supportive care needs compared to patients suffering from other types of cancer ($\chi^2(4)$=9.861, p-value=0.031).

Differences in instruments scores with respect to comorbidities (Data not shown in Tables S4-8):

- Significant distress differences (SAQ-P) were observed in patients suffering from chronic diseases (mean rank: 51.14 vs. 33.83, Mann-Whitney U=328.00, p=0.036), compared to those free of chronic disease, suggesting that chronic diseases are associated with higher rates of significant distress.
- Significant differences in concerns during the last month (CC-LM) were observed between patients suffering from chronic diseases (mean rank 51.15 vs. 33.79, Mann-Whitney U=327.5, p=0.042) compared to those free of disease, suggesting that chronic diseases are associated with higher concerns compared to those free of chronic disease.
- Significant differences in concerns during the last week (CC-LW) were observed between patients suffering from chronic diseases (mean rank 51.22 vs. 33.29, Mann-Whitney U=321.5, p=0.037) compared to those free of disease, suggesting that chronic diseases are risk factors for increased concerns even the last week.
- No significant differences in supportive care needs (NEQ) were observed between patients suffering from chronic diseases (mean rank 49.52 vs. 45.33, U=466.00, p=0.633) compared to those free of disease, suggesting that chronic diseases are not associated with the increased number of supportive care needs.
